# Reorienting Allied Health Into Community‐Based Care for People Experiencing Trauma and Social Disadvantage

**DOI:** 10.5694/mja2.70251

**Published:** 2026-07-20

**Authors:** Simon Rosenbaum, Grace McKeon, Gülşah Kurt, Oscar Lederman, Kemi Wright, Sabuj Kanti Mistry, Jackie E. Curtis, Philip B. Ward, Zachary Steel, Hamish Fibbins, Rachel Morell, Melissa C. Eaton, Andrew Watkins, Ben Harris‐Roxas, Brendan Goodger, Eleanor Beck, Megan Teychenne, Joseph Firth, Davy Vancampfort, David Burns, Russell Roberts, Tristan Favaloro, Danielle Weber, Rosanna Barbero, Vasili Maroulis, Melissa Holmes, Stefan Mackenzie, Chiara Mastrogiovanni, Afsana Anwar, Uzma Choudhry, Catherine Sherrington, Jane Currie, Thomas Gadsden, Scott Teasdale, For the Nutrition, Exercise and Social Equity (NExuS) Consortium

**Affiliations:** ^1^ Discipline of Psychiatry and Mental Health, Faculty of Medicine and Health University of New South Wales Sydney New South Wales Australia; ^2^ School of Psychology, Faculty of Science University of New South Wales Sydney New South Wales Australia; ^3^ School of Sport, Exercise and Rehabilitation, Faculty of Health University of Technology Sydney Sydney New South Wales Australia; ^4^ School of Health Sciences, Faculty of Medicine and Health University of New South Wales Sydney New South Wales Australia; ^5^ School of Population Health University of New South Wales Sydney New South Wales Australia; ^6^ Mindgardens Neuroscience Network Sydney New South Wales Australia; ^7^ School of Medical, Indigenous and Health Sciences, Faculty of Science, Medicine and Health University of Wollongong Wollongong New South Wales Australia; ^8^ Primary Care Improvement Central and Eastern Sydney PHN Mascot New South Wales Australia; ^9^ Institute for Physical Activity and Nutrition (IPAN), School of Exercise and Nutrition Sciences Deakin University Melbourne Victoria Australia; ^10^ Division of Psychology and Mental Health The University of Manchester Manchester UK; ^11^ Department of Rehabilitation Sciences KU Leuven Leuven Flanders Belgium; ^12^ University Psychiatric Center KU Leuven Leuven Flanders Belgium; ^13^ Collective Leisure Sydney New South Wales Australia; ^14^ Charles Sturt University Bathurst New South Wales Australia; ^15^ Body Focused Team, NSW Service for the Treatment and Rehabilitation of Torture and Trauma Survivors Fairfield New South Wales Australia; ^16^ Refugee Health Access, NSW Refugee Health Service Sydney New South Wales Australia; ^17^ Addison Road Community Centre Organisation Marrickville New South Wales Australia; ^18^ Marrickville Legal Centre Marrickville New South Wales Australia; ^19^ Stepping Out Sydney New South Wales Australia; ^20^ Mission Australia Sydney New South Wales Australia; ^21^ Sydney Musculoskeletal Health University of Sydney Sydney New South Wales Australia; ^22^ Discipline of Nursing, School of Population Health, Faculty of Medicine and Health University of New South Wales Sydney New South Wales Australia; ^23^ The George Institute for Global Health University of New South Wales Sydney New South Wales Australia; ^24^ School of Psychiatry University of New South Wales Sydney New South Wales Australia

**Keywords:** allied health occupations, diet, exercise physiology, health promotion, mental health services, physiotherapy, prevention and control

## Abstract

Trauma and social disadvantage are strongly associated with higher rates of chronic disease, partly driven by modifiable risk factors such as physical inactivity and poor diet. Despite strong evidence supporting exercise and dietary interventions for both physical and mental health, access to allied health professionals—particularly exercise physiologists, physiotherapists and dietitians—remains profoundly inequitable. Current prevention efforts predominantly reach individuals with stable living conditions and sufficient resources, ultimately privileging the privileged and entrenching health disparities. To close these gaps, these workforces must be reoriented: embedded within trusted community settings and delivered earlier in the care pathway, in ways that are trauma‐informed and responsive to social context.

## 1 Introduction

Trauma and social disadvantage are major drivers of health inequity in Australia, contributing to disproportionate rates of chronic disease, multimorbidity and psychological distress [1]. Much of this burden is linked to physical inactivity and poor diet, two powerful yet modifiable determinants of health. Interventions targeting these behaviours are among the few strategies that improve both physical [2, 3] and mental [4] health and are recommended in Australian and international guidelines for the management, treatment and prevention of chronic conditions, including depression [5, 6], cardiometabolic disease and obesity [2]. However, the effectiveness of these interventions is shaped by the social and economic conditions in which people live, including access to nutritious food, safe environments, and the time and resources needed to engage in health‐promoting behaviours. Access to allied health professionals trained to deliver such interventions remains profoundly inequitable. In 2022–2023, fewer than 1% of Australians accessed exercise physiology or dietetic services, and individuals in the lowest socio‐economic quintile received 40% fewer services than individuals in the highest [7]. People experiencing trauma and social disadvantage (including refugees and asylum seekers, survivors of sexual and gendered violence, people facing homelessness or food insecurity) are among those least likely to benefit from these preventive strategies [8, 9, 10]. These inequities reflect not only differential access to care but also the limitations of clinic‐based, referral‐driven models that are often inaccessible or unacceptable to those with complex social needs. This mismatch entrenches health gaps and raises a pressing question: how can these allied health services be delivered earlier and within trusted community settings, in ways that are trauma‐informed and responsive to the social conditions that shape engagement?

## 2 Why Reorienting Allied Health Services Matters?

Exercise and dietary interventions are among the few strategies that improve both physical and mental health. Their benefits extend across conditions including depression, obesity, diabetes, and cardiovascular disease, and the interventions are endorsed as first‐line or adjunctive care in both Australian and international guidelines [1, 2, 11]. Yet, despite the strength of the evidence, translation into practice remains uneven. The third report of the *Lancet Psychiatry Physical Health Commission* [12], published in August 2025, shifts the focus from why programs work, to how they can be equitably and sustainably embedded within routine care. This imperative is echoed in the World Health Organization (WHO) 2025 *Guidance on mental health policy and strategic action plans* [13], which frames exercise and dietary interventions as rights‐affirming, person‐centred and community‐embedded, with outcomes extending beyond symptom reduction to enhancement of dignity, autonomy, resilience, hope and social connection. Demand for mental health and primary care continues to rise [14, 15], while bulk‐billing declines and wait times increase [16], placing growing pressure on existing systems. Together, these trends create a window of opportunity to reorient allied health services towards prevention, community settings and populations currently underserved.

The challenge of reorienting allied health services earlier and closer to community settings is particularly urgent in Australia. Although Australia has a highly trained allied health workforce including over 8000 Accredited Exercise Physiologists (AEPs) and 9000 Accredited Practising Dietitians (APDs), these professionals remain concentrated in private, clinic‐based models outside of the acute sector [17]. Access to care is shaped by financial barriers, Medicare rebates, out‐of‐pocket costs and private insurance. In addition, trauma and related psychosocial factors can contribute to distrust, avoidance of healthcare settings and difficulties engaging with treatment [18], further limiting access for people experiencing disadvantage. For these populations, the combined impact of economic constraints and trauma‐related challenges renders evidence‐based, preventive care largely inaccessible.

Unless allied health services are reoriented earlier and closer to community settings, that is upstream in the care pathway, and delivered outside traditional clinic‐based models, prevention will remain a privilege [19], not a right. Australia has both the workforce and the evidence; the challenge is reconfiguring systems to deliver these interventions where they are needed most.

## 3 Translating Allied Health Innovations From Clinical to Community Settings

Several recent innovations demonstrate how allied health models developed within clinical services can be adapted and embedded within community settings to improve access and equity. One such example is Keeping the Body in Mind (KBIM) [20, 21, 22], developed in south‐east Sydney, which integrates exercise physiology and dietetics into an early psychosis program with a specific focus on preventing antipsychotic‐induced weight gain. It improved physical health outcomes and has since been adapted in other mental health settings. KBIM proved that lifestyle interventions can become part of core business rather than optional extras when allied health is embedded in the treatment plan.

Building on KBIM's success in clinical settings, the Addi Moves initiative [23] illustrates how allied health can be embedded in community contexts. Delivered in partnership with the Addison Road Community Organisation in inner west Sydney, it provides free, trauma‐informed exercise physiology primarily to women and families affected by displacement, violence and social disadvantage [23]. Sessions are flexible in timing and format, and delivered in safe, supportive environments that foster trust and respect participants' lived experiences. The program also includes child‐friendly spaces, allowing women to attend with their children and fully engage in the activities. Preliminary evaluation indicates high engagement and consistent improvements in mood following sessions, with no reported serious adverse events. Addi Moves is sustained through a structured pipeline of student placements under professional supervision and reaches people already engaged with Addison Road's food relief and community programs (which support around 9000 people each week) and is now expanding to integrate nutrition and dietetic support through Addi Eats. This partnership model ensures services reach those most in need, rather than relying on referral into traditional clinical systems.

Another innovation developed in a clinical setting is Keeping Our Staff in Mind (KoSiM) [24, 25], a workforce‐focused model that enables clinical and non‐clinical frontline staff in mental health services to experience lifestyle interventions themselves. This approach builds confidence, capacity and cultural change before staff are asked to deliver or refer. Its primary impact lies in embedding lifestyle care as a sustainable element of routine practice. Although KoSiM is situated within clinical services, it demonstrates how workforce‐focused approaches can support a shift towards prevention and lifestyle‐oriented care within existing systems and can be adapted to community‐based contexts. This aligns with the WHO call to expand the mental health workforce through task‐shifting, enabling a broader workforce to contribute to trauma‐informed, rights‐based care. Similar workforce‐focused principles have also been applied within community settings, including Addi Moves. Together, these initiatives demonstrate that reorienting allied health services earlier and closer to community settings is both feasible and impactful. By embedding allied health within trusted services, co‐designed with communities and supported by innovations such as student placements and reflective supervision, these models extend prevention beyond traditional clinic‐based care into the settings where people experiencing trauma and disadvantage already seek support.

## 4 Reorienting Allied Health for Equity

To achieve sustainable and equitable reorientation of allied health services, several structural and workforce strategies must be considered. Reorienting allied health requires locating services where people already are, rather than expecting those most affected by trauma and disadvantage to navigate complex referral systems. Partnerships with trusted organisations including food pantries, homelessness services, refugee health programs and sexual violence support services, provide direct pathways to populations who stand to benefit most from preventive care. Embedding lifestyle‐based allied health services within these contexts can help ensure interventions are delivered in safe, culturally responsive environments, alongside the supports people are already accessing. It also creates opportunities for the specialised staff within these services to mentor and support allied health students. These specialised staff hold unique expertise in working with hard to reach populations who may otherwise be less likely to engage in help seeking behaviour. This reciprocal process generates new knowledge and ways of working, rather than simply transplanting existing models of care. In Seye Abimbola's terms, it ‘connects a system to more of itself’, [26] strengthening the links between health and community in mutually reinforcing ways.

## 5 Student Placements as a Workforce Strategy

A critical enabler of this approach is the student placement pipeline [27, 28]. Exercise physiology, dietetics and physiotherapy programs produce thousands of graduates each year, yet opportunities to train in community and trauma‐informed settings remain limited. Structured placement models, such as those used in Addi Moves, allow students to contribute to service delivery under professional supervision while gaining invaluable experience in equity‐focused practice. Emerging evidence suggests that allied health placements can also support workforce capacity by strengthening service delivery, staff capability and care quality [28]. This experience helps cultivate practitioners who are socially aware, engaged and better prepared for work in settings shaped by trauma and disadvantage. The dual benefit of expanding access while building the next generation of practitioners offers a sustainable and scalable workforce solution.

Relationships with frontline community organisations are equally important. By working alongside services already trusted by people experiencing disadvantage, allied health can be normalised as part of holistic support rather than an additional hurdle. In this way, prevention becomes accessible, acceptable and integrated into daily life. Reorienting allied health closer to community settings is therefore not about creating parallel systems, but about embedding evidence‐based interventions into the very services that already sustain communities. This may also include community‐based supports accessed through individualised funding models, including for some people with National Disability Insurance Scheme (NDIS) support packages, although access will depend on whether these services are visible, navigable and supported within existing care and funding systems. Student pipelines and service partnerships together provide a practical roadmap for shifting lifestyle care out of clinics and into the spaces where it is most urgently needed.

Achieving this reorientation will require coordinated action across multiple levels of the health system. Policy makers and funders must support commissioning and reimbursement models that enable allied health services to be delivered within community settings, rather than relying solely on clinic‐based, referral‐driven care. This may require moving beyond fee‐for‐service approaches towards funding models that support prevention, continuity of care and multidisciplinary delivery. In parallel, universities and training providers must expand placement opportunities in community and trauma‐informed settings to build a workforce equipped to deliver equitable care.

Current allied health service models in Australia largely suit those with stability and resources. It is time to re‐imagine what prevention‐focused allied health care is, who it is for and where it should be delivered. Rather than clinic‐based extras for the already well served, exercise and dietary support must be seen as essential, rights‐based care, aligned with the WHO call for rights‐based, holistic and community‐integrated systems. This means moving prevention into community spaces where people affected by trauma and disadvantage already seek support, building reciprocal partnerships with trusted organisations that sustain communities and training a workforce equipped for equity through student pipelines and culturally responsive practice. Unless allied health is reoriented to engage earlier and closer to community settings—upstream in the care pathway—prevention will remain a privilege of the few.

## Author Contributions

Conceptualisation: S.R., G.M., G.K., O.L., K.W., S.K.M., J.E.C., P.B.W., Z.S., H.F., R.M., M.C.E., A.W., B.H.‐R., B.G., E.B., M.T., J.F., D.V., C.M., A.A., U.C., C.S., J.C., T.G. and S.T. Writing, review and editing: all authors.

## Funding

S.R. is funded by a National Health and Medical Research Council (NHMRC) EL2 fellowship (APP2017506). M.T. is supported by an NHMRC Emerging Leadership Fellowship (APP1195335). S.T. is funded by an NHMRC EL1 fellowship (APP2017302). The contents of the published material are solely the responsibility of the individual authors and do not reflect the views of the funding bodies.

## Disclosure

Not commissioned; externally peer reviewed.

## Conflicts of Interest

S.R. has received honorarium from a co‐edited book on exercise and mental illness (Elsevier).

## Data Availability

The authors have nothing to report.
